# Role of Magnetic Resonance Venography in the Evaluation of Cerebral Veins and Sinuses Occlusion

**DOI:** 10.7759/cureus.84719

**Published:** 2025-05-24

**Authors:** Nihar Doggalli, Ravi Kumar Yeli, Siddaroodha Sajjan, Suresh Kanamadi, Vishal Nimbal

**Affiliations:** 1 Department of Radiology and Imaging, Shri B M Patil Medical College, Hospital and Research Centre, Bijapur Lingayat District Educational Association (BLDE) (Deemed to be University), Vijayapura, IND; 2 Department of Radiology, Shri B M Patil Medical College, Hospital and Research Centre, Bijapur Lingayat District Educational Association (BLDE) (Deemed to be University), Vijayapura, IND; 3 Department of Radiodiagnosis, Shri B M Patil Medical College, Hospital and Research Centre, Bijapur Lingayat District Educational Association (BLDE) (Deemed to be University), Vijayapura, IND

**Keywords:** cerebral venous sinus thrombosis (cvst), diffusion-weighted imaging, edema, mr venography, subarachnoid hemorrhage

## Abstract

Objective

This study aims to evaluate the diagnostic efficacy of various imaging techniques in identifying cerebral venous thrombosis (CVT) and to investigate the clinical features associated with this condition.

Methodology

This prospective study, conducted from September 2022 to June 2024 at Shri B.M. Patil Medical College, included 55 patients clinically suspected of having CVT. Magnetic resonance imaging (MRI) and magnetic resonance venography (MRV) were used for diagnosis, employing sequences such as axial T1-weighted spin echo (T1 SE), sagittal T1 fluid-attenuated inversion recovery (T1 FLAIR), axial and coronal T2 fast spin echo (T2 FSE), axial T2 star-weighted gradient echo (T2*), and two-dimensional time-of-flight (2D TOF) imaging. Diffusion-weighted imaging (DWI) was utilized to distinguish between cytotoxic edema, indicating cellular injury typically resulting from infarction, and vasogenic edema, which reflects fluid leakage due to blood-brain barrier disruption. The study assessed the clinical features and imaging findings and compared the diagnostic efficacy of MRV to that of conventional MRI. The presence of subarachnoid hemorrhage (SAH) as an indicator of CVT was also evaluated. Chi-square tests were used to determine statistical significance, with a p-value of <0.05 considered significant.

Results

Of the 55 patients, 36 (65.5%) were men and 19 (34.5%) were women. There was no significant difference between MRV and T1w/FLAIR in detecting thrombosis of the superior sagittal sinus (34 (61.8%)), left transverse sinus (17 (30.9%) vs. 13 (23.6%), p=0.3918), right transverse sinus (26 (47.3%) vs. 26 (47.3%)), right sigmoid sinus (6 (10.9%) vs. 6 (10.9%)), and left sigmoid sinus (20 (36.4%) vs. 20 (36.4%)). CVT was identified more frequently by MRV (9 (16.3%)) than by T1w/FLAIR (3 (5.4%)), with a p-value approaching significance (p=0.0565). Thrombosis in deep venous segments was detected more often by MRV (12 (21.8%)) compared to T1w/FLAIR (two (3.6%)), with a statistically significant difference (p=0.0104).

Conclusion

The study underscores the importance of advanced imaging techniques, including MRV and DWI, in accurately diagnosing CVT. Distinguishing between cytotoxic and vasogenic edema is critical for prognosis. SAH may be a valuable diagnostic clue for CVT, especially when the basal cisterns are unaffected. Despite its limitations, T2* imaging aids in detecting specific aspects of CVT. These findings highlight the need for comprehensive imaging strategies to enhance diagnostic accuracy and improve patient management. However, limitations such as the relatively small sample size and single-center design may affect the generalizability of the results. Larger multicenter studies are warranted to validate these observations.

## Introduction

Cerebral venous occlusion (CVO) refers to a condition in which an intraluminal blockage caused by cerebral venous thrombosis (CVT) or external compression obstructs venous blood flow in the brain [1]. Although it is less prevalent than arterial disease as a cause of cerebral infarction, its potential morbidity makes it a significant consideration [1]. Cerebral venous occlusive disease is an elusive and frequently misdiagnosed cause of abrupt neurological deterioration [2]. Because clinical symptoms and signs are often non-specific, imaging is essential for diagnosing this disorder [2]. The precise incidence of CVT remains uncertain due to the lack of rigorously conducted epidemiological studies in the current literature. Research indicates that the mortality rate ranges from 5% to 15% [3]. CVT affects individuals across all age groups, presents with a broad spectrum of clinical manifestations, and has diverse predisposing factors that collectively make diagnosis particularly challenging [3]. In all neuroradiological evaluations, the diagnostic checklist should include an assessment for signs of CVT [4].

The cerebral venous system has been evaluated using various radiological techniques, including conventional and digital subtraction cerebral angiography, computed tomography (CT), magnetic resonance imaging (MRI), magnetic resonance venography (MRV), and CT venography. These modalities have significantly improved the detection of venous pathologies [5]. The gold standard for diagnosing cerebral venous sinus thrombosis (CVST) is cerebral angiography [6]. However, even targeted injections into the internal or common carotid arteries may fail to opacify the dural sinuses in some instances. Angiography carries a 2% risk of significant complications and mortality, is invasive, is time-consuming, and may result in complications at the catheter insertion site [6]. Conventional CT techniques tend to underestimate the extent and severity of venous infarcts and sinus involvement, potentially missing CVST in 16%-40% of children and adults [7,8].

Moreover, CT can yield false-positive results. While CT venography is comparable in diagnostic reliability to MRI for cerebral sino-venous thrombosis, it has limitations, including the need for intravenous contrast and significant exposure to ionizing radiation [5]. Additionally, CT is unsuitable for repeated follow-ups or for use as a screening tool in pregnant patients [5].

Over the past decade, MRI has proven to be an effective alternative. When combined with MR venography techniques, MRI has rapidly become the preferred modality for diagnosing and evaluating CVT and dural sinus thrombosis in settings where MRI facilities are available [7]. Time-of-flight (TOF) MR venography is the most commonly used technique for diagnosing CVT [8]. The current gold standard for evaluating CVT is the integration of MRI with MRV, which enables accurate diagnosis and localization of thrombosis [9].

This study aims to assess the role of MRI and MRV in the early diagnosis of CVST, with a focus on advanced sequences such as fluid-attenuated inversion recovery (FLAIR) and diffusion-weighted imaging (DWI) to detect parenchymal changes and distinguish between cytotoxic and vasogenic edema. By correlating MR venographic findings with clinical features and analyzing apparent diffusion coefficient (ADC) values, the study seeks to identify imaging markers that may guide prognosis and therapeutic decisions. It also examines thrombosis patterns and residual effects of CVST on brain parenchyma. Ultimately, this research hypothesizes that integrating MRI and MRV techniques will enhance diagnostic accuracy and contribute to improved clinical management strategies for patients with suspected CVT.

## Materials and methods

Study design and setting

This hospital-based prospective study was conducted at Shri B M Patil Medical College, Vijayapura, focusing on patients referred to the Department of Radiology who met the inclusion criteria. The study period was from September 2022 to June 2024 and included a sample size of 55 cases. It commenced following approval from the Institutional Ethics Committee (Approval No. BLDE (DU)/IEC/759/2022-23).

Selection criteria

The selection criteria included young and middle-aged patients of both sexes who presented with clinically suspected CVT and underwent MRV and MRI brain sequences, including fluid-FLAIR and DWI. Clinically suspected CVT was defined as patients presenting with one or more of the following features: severe or progressive headache, seizures, papilledema, focal neurological deficits, altered consciousness, or signs of raised intracranial pressure, as evaluated by the attending neurologist or emergency physician. Clinical features assessed included headache, visual disturbances, seizures, altered consciousness, and neurological deficits.

Participants who presented with symptoms but had negative imaging findings were closely monitored, and follow-up assessments were conducted to evaluate any changes in clinical status. Exclusion criteria included individuals with cochlear implants, pacemakers, artificial heart valves, or any metallic implants in their bodies and those with a history of claustrophobia or a known history of previous CVT currently under follow-up.

Data source and variables

The imaging protocol for this study utilized a 1.5 Tesla GE SIGNA MRI machine (GE Healthcare, Chicago, IL, USA) to evaluate each subject comprehensively. The MRI sequences included axial T1-weighted spin echo (T1 SE), sagittal T1-weighted fluid-attenuated inversion recovery (T1 FLAIR), axial and coronal T2-weighted fast spin echo (T2 FSE), axial FLAIR, axial T2 star-weighted gradient echo (T2*), and two-dimensional time-of-flight (2D TOF) magnetic resonance angiography. These sequences were selected to provide a detailed assessment of the brain's structural and vascular characteristics, which are critical for diagnosing CVST.

The imaging process began with acquiring axial T1 SE images, which provided high-resolution anatomical details. Sagittal T1 FLAIR sequences were used to suppress fluid signals, improving the visibility of lesions and abnormalities in the brain parenchyma. Axial and coronal T2 FSE sequences were included to provide contrast information, which was particularly useful for identifying edema or other pathologies. Axial FLAIR sequences further aided in detecting hyperintense lesions by suppressing cerebrospinal fluid signals, while axial T2* sequences were beneficial for identifying hemorrhages and calcifications. The 2D TOF sequence in the sagittal plane was employed to visualize blood flow within the cerebral venous system without using contrast agents. Following the 2D TOF imaging, the original images were carefully reconstructed into three-dimensional (3D) maximum intensity projection (MIP) images. This reconstruction process enhanced visualization of the venous anatomy and facilitated the identification of thrombotic occlusions or other abnormalities. The sequence parameters applied are detailed in Table 1.

**Table 1:** Imaging protocol TE: echo time TR: repetition time FOV: field of view MM: millimeters NEX: number of excitations Axial T1: axial T1-weighted spin echo Axial T2: axial T2-weighted fast spin echo Axial flair: axial T1-weighted fluid-attenuated inversion recovery Coronal T2: coronal T2-weighted fast spin echo Axial T2*: axial T2*-weighted imaging DWI: diffusion-weighted imaging 2D TOF: two-dimensional time-of-flight

Sequence	TE (ms)	TR (ms)	FOV (cm)	Slice Thickness (mm)	Matrix	NEX
Axial T1	9.5	700	261 x 261	5	320 x 206	0.5
Axial T2	102.9	4913	260 x 260	5	256 x 192	1
Axial FLAIR	92.9	8262	261 x 261	5	320 x 224	1
Coronal T2	106.8	5914	230 x 230	5	320 x 224	1
Axial T2*	92.9	620	261 x 261	5	256 x 192	0.75
DWI	99.7	5131	260 x 260	5	120 x 140	4
2D TOF	5.6	12.3	250 x 250	5	320 x 192	0.6

Two board-certified radiologists with over five years of experience in neuroimaging independently reviewed all the imaging studies. Both reviewers were blinded to clinical information and each other’s findings. Discrepancies in interpretation were resolved by mutual consensus.

Classification of imaging findings

Parenchymal abnormalities were categorized as hemorrhagic infarction, non-hemorrhagic infarction, or edema. Edema was further classified as vasogenic or cytotoxic based on signal characteristics on DWI and ADC maps. Vasogenic edema was defined by hyperintensity on DWI with elevated ADC values, while cytotoxic edema was identified by DWI hyperintensity with reduced ADC values. ADC values were analyzed by manually placing circular regions of interest (ROIs) within areas of signal abnormality, and the mean ADC value was recorded. Measurements were taken across three consecutive slices and averaged. These values were compared with normal contralateral brain regions to confirm the nature of edema.

Statistical analysis

The statistical analysis in this study was conducted using the chi-square test to determine the significance of associations between categorical variables. IBM SPSS for Statistics version 23.0 (IBM Corp, Armonk, NY, USA) was used for statistical analysis. Data were presented as numbers and percentages for demographic characteristics, causes, symptoms, parenchymal changes, presence of collateral venous pathways, and imaging detection methods. The chi-square test was applied to compare the detection rates between MRV and T1-weighted fluid-attenuated inversion recovery sequences for various sinus involvements. Statistical significance was assessed using a p-value of less than 0.05, with specific results reported where applicable.

## Results

Table 2 describes the demographic distribution of CVT patients. Of the 55 patients, 36 were men (65.5%), while 19 were women (34.5%). The age distribution of the patients showed that 18 (32.7%) were aged between 20 and 29 years, followed by 14 (25.5%) in the 30-39 age group. Eight patients were aged 40-49 (14.5%) and five patients were aged 50-59 (9.1%). Three patients (5.5%) constituted the youngest and oldest age groups, those under 20 years and those aged 60-69 years, while four (7.3%) patients were 70 years and above.

**Table 2:** Demographic distribution of cerebral venous thrombosis (CVT) patients

Category	Number of Patients	Percentage (%)
Sex		
Female	19	34.5
Male	36	65.5
Total	55	100
Age (in years)		
<20	3	5.5
20-29	18	32.7
30-39	14	25.5
40-49	8	14.5
50-59	5	9.1
60-69	3	5.5
70+	4	7.3
Mean Age (years)	38.45	
Standard Deviation	18.35	

Table 3 illustrates the risk factors of CVT among the patients. The leading cause was alcohol consumption, observed in 13 (23.6%) patients. Unknown causes also accounted for 13 (23.6%) of cases. Postpartum CVT was identified in eight (14.5%) of the patients, while dehydration and sepsis each contributed to five (9.1%) cases. Trauma was the cause in four (7.3%) patients. Other causes included hypertension and oral contraceptives, each accounting for two (3.6%) cases, and less common causes, such as demyelinating disorders, diarrhea, and mastoiditis, each accounted for one (1.8%) case.

**Table 3:** Risk factors of cerebral venous thrombosis (CVT)

Cause	Number of Patients (N=55)	Percentage (%)
Alcohol	13	23.6
Dehydration	5	9.1
Demyelinating Disorder	1	1.8
Diarrhea	1	1.8
Hypertension	2	3.6
Mastoiditis	1	1.8
Oral Contraceptives	2	3.6
Postpartum	8	14.5
Sepsis	5	9.1
Trauma	4	7.3
Unknown	13	23.6

Table 4 details the symptoms experienced by CVT patients. The most common symptom was a headache, reported by 30 (54.5%) patients. Seizures were present in 18 (32.7%) cases, while altered sensorium was observed in 15 (27.3%) patients. Neurological deficits, defined as impairments in motor or sensory function, were reported by 7 (12.7%) patients and included specific symptoms such as blurring of vision (three patients) and loss of consciousness (five patients). Additionally, nine (16.4%) patients experienced giddiness. Other symptoms included vomiting in two (3.6%) patients and fever in one (1.8%) patient.

**Table 4:** Symptoms of cerebral venous thrombosis (CVT)

Symptom	Number of Patients	Percentage (%)
Headache	30	54.54
Neurological Deficit (impairments in motor or sensory function)	7	12.72
Seizures	18	32.72
Loss of Consciousness	5	9.09
Altered Sensorium	15	27.27
Vomiting	2	3.63
Fever	1	1.81
Giddiness	9	16.36
Blurring of Vision	3	5.45

Table 5 presents the parenchymal changes observed in CVT patients. Hemorrhagic infarction (HI) was noted in 28 (50.9%) patients, making it the most common parenchymal change. Non-hemorrhagic infarction was observed in five (9.1%) patients. Meanwhile, 21 (38.1%) patients had normal parenchymal findings without any infarction.

**Table 5:** Parenchymal changes in cerebral venous thrombosis (CVT) patients

Parenchymal Change	Number of Patients	Percentage (%)
Hemorrhagic Infarction (HI)	28	50.9
Non-Hemorrhagic Infarction	5	9.1
Normal	22	40

Table 6 describes the presence of collaterals and subarachnoid hemorrhage (SAH) in CVT patients. Collaterals were present in eight (14.5%) patients, while the remaining 47 (85.5%) did not exhibit collaterals. Regarding SAH, it was detected in five (9.1%) patients, whereas 50 (90.9%) did not present with SAH.

**Table 6:** Presence of collaterals and subarachnoid hemorrhage (SAH)

Category	Number of Patients	Percentage (%)
Collaterals		
Yes	8	14.5
No	47	85.5
Total	55	100
SAH (Subarachnoid Hemorrhage)		
Yes	5	9.1
No	50	90.9
Total	55	100

Table 7 illustrates the distribution of focal abnormalities by edema type in the study population. Among the 55 patients with focal abnormalities, 11 (20.0%) had co-existing cytotoxic and vasogenic edema. Cytotoxic edema alone was present in two (3.6%) cases, while 35 (63.6%) patients showed no edema. Vasogenic edema was observed in seven (12.7%) patients.

**Table 7:** Distribution of focal abnormalities by edema type

Edema Types	Number of Patients	Percentage (%)
Co-existing Edema	11	20
Cytotoxic Edema	2	3.6
No Edema	35	63.6
Vasogenic Edema	7	12.7
Total	55	100

Table 8 provides information on the detection of CVT by imaging techniques, comparing MRV with T1w/FLAIR. The superior sagittal sinus was detected in 34 (61.8%) patients by MRV and T1w/FLAIR, showing no significant difference. The left transverse sinus was detected by MRV in 17 (30.9%) patients and by T1w/FLAIR in 13 (23.6%) patients, with a chi-square value of 0.7331 and a p-value of 0.3918, indicating no significant difference. Both techniques detected the right sigmoid and right transverse sinuses equally in six (10.9%) and 26 (47.3%) patients. Both methods detected the left sigmoid sinus in 20 (36.4%) patients. CVT was detected more frequently by MRV in 9 (16.3%) patients than by T1w/FLAIR in 3 (5.4%) patients, with a chi-square value of 3.367 and a p-value of 0.0565, approaching significance. Thrombosed deep venous segments were significantly more detected by MRV in 12 (21.8%) patients than by T1w/FLAIR in two (3.6%) patients, with a chi-square value of 9.131 and a statistically significant p-value of 0.0104.

**Table 8:** Detection of cerebral venous thrombosis (CVT) by imaging techniques

Sinus Involved	Detected by MRV	Detected by T1w/FLAIR	Chi-Square (X²)	P-Value
Superior Sagittal Sinus	34 (61.8%)	34 (61.8%)	0	1
Left Transverse Sinus	17 (30.9%)	13 (23.6%)	0.7331	0.3918
Right Sigmoid Sinus	6 (10.9%)	6 (10.9%)	0	1
Right Transverse Sinus	26 (47.3%)	26 (47.3%)	0	1
Left Sigmoid Sinus	20 (36.4%)	20 (36.4%)	0	1
Cortical Venous Thrombosis	9 (16.3%)	3 (5.4%)	3.367	0.0565
Thrombosed Deep Venous Segments	12 (21.8%)	2 (3.6%)	9.131	0.0104

## Discussion

CVT is a challenging condition to diagnose and is frequently associated with sudden neurological deterioration. Diagnosing CVT is difficult due to its broad spectrum of clinical manifestations, various risk factors, and occurrence across different age groups. In this study, several specific risk factors were identified among the included patients, including alcohol consumption, postpartum status, dehydration, and a history of trauma. These factors highlight the diverse etiological landscape of CVT and emphasize the need for clinicians to maintain a high index of suspicion, particularly in populations with identifiable risk factors [3]. Given the non-specific clinical signs and symptoms, imaging plays a crucial role in diagnosis. The current gold standard is the integration of MRI and MRV, which facilitates the precise identification of CVT. MRV is the most effective non-invasive technique for assessing the cerebral venous network [9]. Our findings support this diagnostic approach, as we were able to accurately identify thrombosed sinuses and associated parenchymal changes using MRI and MRV in all included cases.

CVT is observed more frequently in women compared to men. Ameri and Bousser reported a female-to-male ratio of 1.29:1 in a study of 110 cases [6]. However, 36 (65.5%) patients in the current study were men, suggesting a slight male preponderance. While CVT can affect individuals of any age, it is most commonly seen in young adults [10]. In the Indian setting, Pillai et al. highlighted CVT as a primary stroke contributor among younger individuals, with an average age of 38.69 years and the most common age group being the 20-29 years range. The mean age at presentation is almost 10 years lower in women than men due to gender-specific risk factors [11]. The current study found that the most common age group for patients was between 20 and 29 years (18 patients, 32.7%). The 30-39 age group came next, with 14 (25.5%) patients falling into this category, with male patients averaging 42.5 years and female patients averaging 29 years. These demographic patterns are important because they highlight the need to consider CVT in younger male patients as well, especially in regions where risk factor profiles may differ.

Alcohol use and unknown causes each accounted for 13 (23.6%) of the CVT cases in the present study, followed by postpartum causes in eight (14.5%) cases, dehydration in five (9.1%), and sepsis in five (9.1%) cases. These findings are consistent with Ameri and Bousser’s study, which found no identifiable cause in 20-25% of the cases [6]. Nagaraj et al. noted that puerperium was the most frequent predisposing factor, with 200 out of 230 cases (86%) of CVT being puerperal [12]. Bousser MG also observed cerebral venous blockage resulting from clot formation in all 110 individuals in their study [13]. Similarly, El Damarawy et al. found intraluminal thrombus in 86.7% of cases, with meningioma-induced external compression observed in 13.3% [1]. In the current study, intraluminal thrombus was present in 53 (96.6%) cases, with one external compression caused by a meningioma. This similarity in thrombus detection rates reinforces the reliability of imaging in confirming CVT and helps compare our local findings with those reported globally. Meanwhile, our results contrast with the findings of Jeffrey et al., who identified solid tumor-induced cerebral sinus occlusion primarily through compression or invasion by dural or calvarial metastases [14]. In a Dutch study, the most common symptoms were headaches (38%), paresis (30%), and focal seizures (34%) [15]. Poon et al. discovered that headaches were the most commonly reported symptom, affecting 75% of patients, followed by seizures in 37%, and motor or sensory disturbances (34%) [4]. These findings align with the current study, where headaches were reported by 30 (54.5%) patients, with seizures observed in 18 (32.7%) instances and neurological impairments in seven (12.7%) cases. The consistent presentation of symptoms such as headache and seizures across studies underlines their diagnostic importance and suggests that early imaging should be considered in such clinical scenarios.

Focal cerebral irregularities were reported in about half of the cases. However, the affected sinus’s location may not correspond to the site of parenchymal alterations [16]. Simonds et al. observed localized edema in 25% of cases, non-hemorrhagic infarction in 40%, and hemorrhagic infarction in 26.7% [17]. Nagaraj et al. reported hemorrhagic infarction in 41.9% of cases, while 51.6% did not present with hemorrhage [12]. In contrast, Khandelwal et al. found hemorrhagic infarcts in 60% of cases and non-hemorrhagic infarcts in 13% [5]. In the current study, hemorrhagic infarctions accounted for 28 (50.9%) of focal brain abnormalities, while non-hemorrhagic infarctions occurred in five (9.1%) cases. These parenchymal changes may result from cerebral bleeding, vasogenic edema, or cytotoxic edema, with both patterns potentially coexisting. The observed distribution of infarctions in our study lies within the ranges reported in earlier literature, supporting the validity of our imaging findings. Both kinds of edema can cause bleeding, and different patterns coexist in the same area. Diffusion-weighted (DW) magnetic resonance images can differentiate between cytotoxic and vasogenic edema, which has significant therapeutic implications [18,19]. Cytotoxic edema typically indicates irreversible ischemic damage, guiding more aggressive interventions to prevent further ischemia.

In contrast, vasogenic edema is often reversible and can respond to treatments like steroids or other anti-edema therapies. Therefore, identifying the dominant type of edema is crucial in tailoring treatment strategies and enhancing patient outcomes. In clinical practice, this distinction helps clinicians make treatment decisions favoring conservative approaches for reversible vasogenic changes and considering escalation for cytotoxic damage. Mullins et al. identified three types of lesions in CVT patients: persistent low-diffusion lesions consistent with cytotoxic edema, resolving low-diffusion lesions in patients with seizure activity, and elevated diffusion lesions consistent with vasogenic edema [20]. The distribution of focal abnormalities by edema type revealed notable findings in the study population. Among the 55 patients with focal abnormalities, 11 (20.0%) had co-existing cytotoxic and vasogenic edema. Cytotoxic edema alone was present in two (3.6%) cases, while 35 (63.6%) patients showed no edema. Vasogenic edema was observed in seven (12.7%) patients. These results demonstrate the heterogeneity of edema patterns in CVT, emphasizing the importance of using advanced imaging techniques to guide clinical management based on lesion type.

The most extensive cohort study on CVT, involving 624 patients, reported the involvement of the superior sagittal sinus in 62% of the cases, straight sinuses in 18%, and transverse sinuses in 44.7% on the left and 41.2% on the right [21]. The current study found that the superior sagittal sinus was involved in 34 (61.8%) of the cases, the right transverse sinus in 26 (47.3%), the left transverse sinus in 17 (30.9%), and the right sigmoid sinus in six (10.9%). In nine (16.3%) of cases, superficial cortical veins were affected, although no isolated cases of superficial cortical vein involvement existed. Collateral vessels were observed in eight (14.5%) of cases. This close match in sinus involvement rates further validates the reliability of our imaging protocol and enhances the generalizability of our results.

Reports of SAH linked to CVT are rare. The precise cause of SAH in CVT remains unclear. Still, it may result from venous hemorrhagic infarct rupture into subarachnoid spaces or from fragile cortical veins bursting due to venous hypertension in dural sinus thrombosis [22]. In the current study, five (9%) cases presented with SAH, all of which were accompanied by parenchymal hemorrhagic infarctions. MRI combined with MRV is the gold standard for diagnosing CVT, enabling precise identification of thrombus locations [9]. Vogl et al. found MR angiography the preferred method for diagnosing and monitoring dural sinus thrombosis [7]. However, the reliability of flow signal intensities in MRI sequences can be affected by flow-related artifacts, necessitating the use of MRV to complement standard MRI in CVT diagnosis. These findings reinforce the clinical value of multimodal imaging in identifying rare complications like SAH, which can otherwise be missed.

Leach et al. noted that the impact of susceptibility artifacts from CVT is predominantly identified in individuals with low-intensity thrombus on T2-weighted imaging within a week of symptom onset, identifiable through gradient-echo imaging [23]. Susceptibility artifacts from the skull base can complicate the diagnosis of isolated CVT (ICoVT) using T1-weighted, T2-weighted, and MRV imaging alone. M. Boukobza et al. highlighted the value of T2-gradient echo imaging in early ICoVT detection [24]. In the current study, cortical vein thrombosis was found in nine (16.3%) of patients, although no solitary cases were identified. T2 imaging detected all cortical vein thromboses, while T1/T2 sequences only identified three (5.4%) cases. T2*/MRV sequences also proved more effective in detecting thrombosed deep vein segments. These findings underscore the importance of T2* imaging sequences for detecting CVT. Nonetheless, because of notable susceptibility artifacts from the adjacent skull, T2* images were not employed to diagnose dural venous sinuses’ thrombosis.

Limitations of the study

This study has several limitations that should be acknowledged. First, the absence of a universally accepted gold standard, such as digital subtraction angiography (DSA), for confirming the diagnosis of CVT may have limited the definitive accuracy assessment of MRI and MRV findings. Second, the retrospective design introduces potential selection and interpretation biases, as imaging and clinical data were reviewed based on available documentation and imaging quality. Although imaging interpretation was performed independently by two experienced radiologists with consensus resolution, some degree of subjectivity may still exist. Third, this was a single-center study conducted at a tertiary care institution, which may affect the generalizability of the findings to broader or community-based settings. Future multicenter studies with larger cohorts and prospective designs are warranted to validate and expand upon these findings.

## Conclusions

This study highlights the prevalence of hemorrhagic infarction as the most common focal parenchymal abnormality in CVT, with non-hemorrhagic infarction being a secondary finding. The differentiation between cytotoxic and vasogenic edema, facilitated by DWI, proves crucial in assessing prognosis, as vasogenic edema, being reversible, should not be classified as a venous infarct. The study also emphasizes the significance of SAH as a potential indicator of CVT, particularly in cases where the basal cisterns are not involved. MRV consistently demonstrated superior performance in detecting thrombosis compared to traditional MRI sequences, reinforcing its role as the gold standard in CVT diagnosis. Additionally, while the T2* sequence was instrumental in identifying CVT, particularly in deep venous and superficial cortical veins, its utility in detecting dural sinus thrombosis was limited due to susceptibility artifacts from the surrounding calvaria. These findings underscore the importance of using advanced imaging techniques to enhance diagnostic accuracy in CVT.
